# A patient-centered network approach to multidisciplinary-guideline development: a process evaluation

**DOI:** 10.1186/1748-5908-9-68

**Published:** 2014-06-04

**Authors:** Elvira ME Den Breejen, Mirrian AHW Hilbink, Willianne LDM Nelen, Tjerk J Wiersma, Jako S Burgers, Jan AM Kremer, Rosella PMG Hermens

**Affiliations:** 1 Department of Obstetrics and Gynecology, Radboud University Nijmegen Medical Center, Internal postal code 114, PO Box 9101, Nijmegen 6500 HB, The Netherlands; 2 Scientific Institute for Quality of Healthcare, Radboud University Nijmegen Medical Center, Nijmegen, The Netherlands; 3 Dutch College of General Practitioners, Utrecht, The Netherlands

**Keywords:** Clinical practice guideline development, Evaluation, Patient involvement, Clinical care pathway, Infertility

## Abstract

**Background:**

Guideline development and uptake are still suboptimal; they focus on clinical aspects of diseases rather than on improving the integration of care. We used a patient-centered network approach to develop five harmonized guidelines (one multidisciplinary and four monodisciplinary) around clinical pathways in fertility care. We assessed the feasibility of this approach with a detailed process evaluation of the guideline development, professionals’ experiences, and time invested.

**Methods:**

The network structure comprised the centrally located patients and the steering committee; a multidisciplinary guideline development group (gynecologists, physicians, urologists, clinical embryologists, clinical chemists, a medical psychologist, an occupational physician, and two patient representatives); and four monodisciplinary guideline development groups. The guideline development addressed patient-centered, organizational, and medical-technical key questions derived from interviews with patients and professionals. These questions were elaborated and distributed among the groups. We evaluated the project performance, participants’ perceptions of the approach, and the time needed, including time for analysis of secondary sources, interviews with eight key figures, and a written questionnaire survey among 35 participants.

**Results:**

Within 20 months, this approach helped us develop a multidisciplinary guideline for treating infertility and four related monodisciplinary guidelines for general infertility, unexplained infertility, male infertility, and semen analysis. The multidisciplinary guideline included recommendations for the main medical-technical matters and for organizational and patient-centered issues in clinical care pathways. The project was carried out as planned except for minor modifications and three extra consensus meetings. The participants were enthusiastic about the approach, the respect for autonomy, the project coordinator’s role, and patient involvement. Suggestions for improvement included timely communication about guideline formats, the timeline, participants’ responsibilities, and employing a librarian and more support staff. The 35 participants spent 4497 hours in total on this project.

**Conclusions:**

The novel patient-centered network approach is feasible for simultaneously and collaboratively developing a harmonized set of multidisciplinary and monodisciplinary guidelines around clinical care pathways for patients with fertility problems. Further research is needed to compare the efficacy of this approach with more traditional approaches.

## Background

Complex multidisciplinary care is often fragmented and suffers from so-called ‘clinical linkage deficiencies’ [1]. To resolve such deficiencies, many published reports and articles have stressed the importance of implementing integrated and patient-centered care by building bridges between the groups involved [2-6]. Clinical practice guidelines (CPGs) are potential tools for facilitating this shift in clinical care. Unfortunately, the quality of the guidelines varies, and their impact on delivering integrated and patient-centered care is still suboptimal [7-12]. Several problems still hinder CPG development and uptake, namely, inadequate management of conflicts of interest (COIs), limited panel composition, lack of patient involvement, and lack of external review [9]. Furthermore, there is still a strong focus on the single clinical aspects of diseases described in the guidelines rather than on ensuring more integrated care for patients, including attention to matters such as patient-centeredness, coordination and continuity of care. Because of all this, the target users feel no affinity with the guidelines, which impedes full implementation [7,8,10].

Clinical networks are defined as collaborative, professionalized structures ranging from fully integrated service delivery systems to informal communities of practice. These networks have previously proven effective in increasing evidence-based practice and improving care models [13,14]. We aimed to resolve the deficiencies in multidisciplinary guideline (MuG) development and to re-center the focus on the patient’s overall clinical journey rather than independent contributions from each specialty or caring function. Thus, we used the network approach in a clinical area of complex multidisciplinary care, namely, fertility care. We developed a harmonized set of one MuG and four monodisciplinary guidelines (MoGs) around patient clinical pathways, including any care needed for infertile couples (such as aftercare and care given by physicians, gynecologists, and/or urologists). We aimed to assess the feasibility of this patient-centered network approach in a detailed evaluation of the process, professionals’ experiences, and the time required.

## Methods

### Setting

The Dutch Organization for Health Research and Development (ZonMw) funded our project, which took place within the Dutch program of Knowledge Quality and Curative Care. The program’s objective was to improve the development of multidisciplinary CPGs in terms of innovation, collaboration, and efficiency.

### Fertility care

Infertility is commonly defined as ‘any form of reduced fertility with prolonged time of unwanted non-conception’,, and it affects approximately 80 million couples worldwide [15,16]. Dutch fertility care takes place on three levels. Physicians provide primary care that includes an initial fertility assessment. A physician can refer couples to a gynecologist in a general hospital (secondary care) or a university hospital (tertiary care). The gynecologist completes the fertility assessment, determines the cause of infertility, and defines a treatment plan. If a relevant male factor is found, the couple may be referred to an urologist. Clinical chemists and embryologists are also involved in assessments and preparation for the use of medically assisted reproductive techniques, one of which is in vitro fertilization (IVF). Because infertility has a high emotional and psychological impact that can interfere with work, psychologists and occupational physicians are regularly involved with the clinical course of the infertility problem.

### The network

Our steering committee, which included five guideline experts, one implementation expert, and one project coordinator, initiated and coordinated the patient-centered network approach to MuG development. A group of gynecologists, physicians, urologists, clinical embryologists, clinical chemists, a medical psychologist, an occupational physician, and two patient representatives from Freya (the Dutch association for people with fertility problems) assembled to collaborate in developing the guidelines in February 2008. Four MoG groups, including participants mainly from single disciplines, and one MuG group convened to develop the guidelines.

An overlap of participants from the same discipline across the groups and the project coordinator facilitated guideline harmonization. The project coordinator was a member of all five groups and gave feedback to the steering committee. The implementation expert assured attention to the future implementation and anticipated any potential barriers to guideline implementation during all development phases. An independent researcher (MS) evaluated the project.

The network structure consisted of three organized layers comprising seven groups: the centrally located infertile patients and the steering committee, the MuG group, and four MoG groups. For our purposes, this network was to produce one patient-centered MuG on infertility and four related, mainly monodisciplinary owned guidelines. These four included a guideline on general infertility for physicians, a guideline on unexplained infertility for gynecologists, a guideline on male infertility for urologists and gynecologists, and a guideline on semen analysis for clinical embryologists.

### Guideline development

#### Managing conflicts of interest (COIs)

Before starting the guideline development, all members of the groups had to declare any COIs and be officially mandated by their societies. The steering committee discussed all COIs. Participants with significant COIs were excluded from discussions or voting on recommendations for which they had COIs.

### Defining the scope and key questions

We explored the care aspects in the clinical pathways of infertile patients. We paid particular attention to improvements that the patients and professionals found necessary. Various methods were used to collect data about the most relevant aspects.

We conducted 12 exploratory interviews with couples facing the spectrum of issues in the main phases of the clinical pathways in fertility care. These phases may include a physician’s initial fertility assessment, a gynecologist’s fertility assessment, treatment with ovulation induction, intrauterine insemination, IVF, and/or intracytoplasmic sperm injection by a gynecologist, a urologist’s care, and aftercare (whether pregnancy occurred or not). The couples were consecutively invited to participate by means of an information letter that they received when they saw a gynecologic resident in a fertility clinic in Nijmegen or Amsterdam. We phoned potential participants—Dutch-speaking couples with fertility problems who reacted positively to the information letter. The selection of couples was random except for their phase in the clinical pathway. The steering committee translated care aspects that the couples said needed improvement into patient-centered key questions.

Two focus groups were conducted among members of the MuG group, including the two patient representatives, and among main target users of the guideline, including four gynecologists, three physicians, and a urologist. The steering committee translated care aspects that the professionals said needed improvement into medical-technical, organizational, and patient-centered key questions. Example questions for these three categories, respectively, are: ‘What are the indications for IVF treatment?’ ‘Which professionals should be part of the treatment team in a university hospital?’ ‘How should patients be informed about adoption?’

All key questions were given a format defined for the MuG. Then the MoG groups addressed the medical-technical questions and the MuG group addressed the organizational and patient-centered questions.

### Elaborating key questions and formulating recommendations

The participants worked in pairs. They used the PICO (patient, intervention, comparison, and outcome) method to define their search strategy, conduct a systematic literature review, select relevant evidence, and summarize this evidence in formatted evidence tables. They rated (scale: 1 to 5) the evidence according to quality criteria adapted from the Center for Evidence-Based Medicine, version 1999 [17]. The pairs of members then formulated one or more conclusions, their considerations, and one or more concept recommendations. A level of evidence (A to D) was given for each recommendation to be discussed in the particular guideline group [17]. The project coordinator was a member of all guideline groups and coordinated the entire process, which was part of a strategy for harmonizing recommendations across the groups. The project coordinator checked the rating of evidence and grading of recommendations for errors. The steering committee discussed and resolved any discrepancies. The implementation expert checked and improved the implementability of the concept recommendations and the guidelines as a whole using the items of the Guideline Implementability Appraisal (GLIA) Tool. An extensive internal review of the CPGs across the groups was used to harmonize the recommendations. Many patients also participated by formulating and prioritizing recommendations for the MuG [18].

We used advertisements and mailings over a period of seven months to invite patients with fertility problems to formulate recommendations via the Dutch online wiki-based tool at http://www.freyawiki.nl[18]. A patient representative and two members of the steering committee including the implementation expert modified and assessed the implementability of the patient recommendations with the GLIA Tool. Then we asked patients to select their top three or five recommendations for each wiki section (General care, General practice care, Gynecologic care, Urologic care, and Laboratory) [18]. The guideline group assessed the eligibility of the final set of patients’ recommendations within the scope of the guideline.

### Integrating the guidelines

The MoGs addressed medical-technical aspects of care that needed improvement: physician care, care for patients suffering from unexplained infertility or male infertility, and fertility care given by clinical embryologists (semen analysis). The definitive MuG described the overall clinical pathway of patients with fertility problems by merging the main medical-technical, patient-centered, and organizational recommendations from the MuG and MoG groups and the prioritized patient recommendations from WikiFreya [18]. The medical-technical recommendations included transitions in fertility care and care alignment. The patient-centered recommendations dealt with respect for patients’ values, preferences, and needs; information, communication, and education; emotional support; partner involvement; and the attitude of the fertility clinic staff. The organizational recommendations addressed coordination and integration of care, physical comfort, transition and continuity of care, access to care, and staff competence and technical skills. All patient recommendations obtained via WikiFreya were classed as Level P (Patient) evidence. To express patients’ input in the guideline, two patient representatives reformulated patient recommendations as: ‘Patients want to….’ The whole project was planned to be completed in 18 months.

### Evaluation

We applied a stepwise process evaluation to the feasibility of this network approach [19]. The primary outcomes were ‘how’ the planned project was actually carried out and ‘how’ participants perceived the process; the secondary outcome was ‘what’ time was invested. We used a mixed-method evaluation including examination of secondary resources (such as project descriptions), interviews with key figures, and a written questionnaire survey among all participants. An independent researcher (MS) conducted the interviews and collected the data.

### Data collection

#### Examination of secondary resources

We collected all the project data from the project descriptions and minutes of meetings to determine whether the project was carried out as planned.

#### Interviews with key figures

We conducted eight semistructured, in-depth, telephone interviews with eight key figures: the chairpersons of the four MoG groups, four members of the MuG group, and the steering committee (one patient representative, two project leaders, and the project coordinator). We asked the interviewees to chronologically describe the guideline development and their activities in the project. We asked for comments on the overall organization, the methodology of the network approach, the methods of patient involvement. Then we asked for suggestions to improve the approach. Full interviews took approximately 30 min each, and they were fully transcribed verbatim.

#### Questionnaire survey

We based our questionnaire on the interview results so that we could assess participant experiences and measure the time needed for the project. The first part of the four-part questionnaire asked about participants’ background characteristics. The second part pertained to participants’ involvement in the preparation and development phase and the time (excluding travel) needed for each of these phases. The third part included five open questions about the network approach (e.g., the methodology and guideline integration), patient involvement, wiki methodology, and patient contributions to the guideline. The fourth part asked participants to describe facilitators of and barriers to the approach and to suggest improvements. All participants received the questionnaire by post, after the draft guidelines were completed. A reminder was sent six weeks later.

### Data analysi**s**

We used SPSS (version 16.0 for Windows, Data Entry 4.0, SPSS, Chicago, Ill.) to perform descriptive statistics (frequencies, medians, and ranges) on participant background characteristics and to analyze the time data. We used Kwalitan (version 5.0, Malden, The Netherlands) to qualitatively analyze the transcripts of the interviews and the free text responses from the second part of the questionnaire [20].

### Project performance

Two authors (MH and RH) identified all activities and categorized them in the preparation phase or the development phase. The preparation phase included composing development groups, managing COIs, identifying care aspects to be improved, and developing WikiFreya. The development phase included attending meetings, formulating key questions, reviewing, selecting and assessing evidence, writing the guideline and formulating recommendations, harmonizing the guidelines, reviewing and revising draft guidelines, and aligning the guidelines with managing WikiFreya. We compared the actual activities with the planned activities and identified differences.

### Time investments

We counted the regular and extra meetings on the attendance lists, and determined the mean meeting participation rates for each group and the steering committee. We calculated the total time the steering committee spent on the preparation phase. We computed the median actual time for the respondent meetings of each development group, total time for extra respondent meetings, and median extra time respondents spent for each development group and for each of the two phases. The total time for meetings for each group, including the project coordinator was assessed as the ‘mean participation rate’ x ‘number of meetings’ x ‘median time investments’. The total extra time needed was defined as the ‘number of participants for each group’ x ‘median extra time spent by each participant’. The values were corrected for non-responders to the questionnaire. We calculated the project coordinator’s extra time separately. Our calculations totaled the time needed for the development phase.

### Experiences

We analyzed the data descriptively, and we used a special framework to analyze open question responses. We developed the special framework from the interview topic guide corresponding to our study objectives, which included experience with the methodology of the network approach, patient involvement, barriers to and facilitators of the approach, and suggestions for improvement [21]. Two researchers (EB and MH) studied the interviews and the open question responses independently. They identified and coded the parts of the interviewees’ responses that were relevant to the study objectives. They then discussed key issues and discrepancies between their results. The key issues were structured with a view to the study objectives. The questionnaire was based on the interviews, so we only present the results of the questionnaire for each study objective, but no information found under the heading ‘Organization’.

## Results

### Participants

Five (four MoG and one MuG) groups were installed in May 2008. None of the members of the groups declared significant COIs, and all of them were officially mandated by their societies. One of the physicians involved in the MuG group dropped out for private reasons two months later. The MuG chairperson, a physician, concurrently fulfilled this role. Overall, 32 participants were involved in the five groups (Table 1). Four participants fulfilled multiple roles: the project coordinator was a member of the steering committee and all five guideline groups; the chairperson of the MoG group for general infertility was a member of the steering committee; one project leader was a member of the steering committee and chairperson of the MuG group; and one member of the MuG group was a member of the steering committee (Table 1).

**Table 1:** Organizations in the guideline development groups

**Professional societies**	**Number of members in development groups**				
	**Multidisciplinary guideline**	**MoG: general infertility**	**MoG: unexplained infertility**	**MoG: male infertility**	**MoG: semen analysis**
**Dutch Association of Obstetrics & Gynecology**	2	1	4	2	1
**Dutch National Organization of General Practitioners**	2	5	1	1	0
**Dutch Urological Association**	1	0	0	2	0
**Dutch Society for Clinical Embryologists**	1	0	0	0	2
**Dutch Society for Clinical Chemistry**	1	0	0	0	1
**Dutch Society of Occupational Physicians**	1	0	0	0	0
**Dutch Association of Psychologists**	1	0	0	0	0
**Dutch Patient Association for Fertility Problems**	2	0	0	0	0
**Number of participants**	11	6	5	5	4
**Total number of participants = 31**^**a**^					

^a^The project coordinator took part in all working groups, which increased the total number of participants by one.

MoG = Monodisciplinary guideline.

All participants received the evaluation questionnaire (one patient representative was unavailable). The response rate was 79% (n = 27). Table 2 summarizes the background characteristics of the respondents. Of the 59% who were experienced in guideline development, 63% were also experienced in MuG development.

**Table 2:** Background characteristics of questionnaire respondents

**27 respondents**	***n *** **(%)**
**Gender**	
Male	14 (52)
Female	13 (48)
**Age in years**	
26–35	4 (15)
36–45	9 (33)
46–55	11 (41)
>55	3 (11)
**Median years of professional experience in fertility care (range)**	9 (1–26)
**Previous experience in guideline development**^**a**^	
Yes	16 (59)
No	11 (41)

MoG = Monodisciplinary guideline, MuG = multidisciplinary guideline.

^a^Experience in guideline development was determined by the authorship of one or more monodisciplinary or multidisciplinary guidelines.

### Guideline development

The project was carried out as planned, except for minor modifications needed to improve the consistency between the concepts of the guidelines. Face-to-face meetings and additional conferences calls were necessary to discuss discrepancies between recommendations concerning the cut-off points for treating infertility. These recommendations were issued by guidelines for male infertility and unexplained fertility. The cut-off points were eventually based on the existing relevant evidence. Further, there was a lack of consensus on some recommendations in the four MoGs for life-style advices (e.g., alcohol and anabolic steroids use) issued in all four MOGs as well as lack of and a lack of underlying evidence. This necessitated two additional consensus meetings, which were attended by four members of the MuG (including the chairperson) and the chairpersons of all MoG groups. These consensus meetings produced overall recommendations for life-style advice based on evidence regarding pre-pregnancy counseling. The MuG group reviewed the drafts of the MoG groups and vice versa. Then, the project coordinator initiated a conference call in order to reach consensus on the recommendations based on the expert opinion of the MoGs. Via WikiFreya, 298 patients formulated 289 recommendations, which 80 patients prioritized into 21 recommendations. These recommendations were included in the definitive guideline [18].

### Resulting guidelines

One MuG and four related MoGs were developed in 20 months; all were written in Dutch:

http://www.nvog-documenten.nl/uploaded/docs/Landelijke%20netwerkrichtlijn%20Subfertiliteit%20def.pdf

http://www.nvog.nl

http://www.nhg.org

http://www.nvu.nl

http://www.embryologen.nl

The definitive MuG follows the overall clinical care pathway for patients with infertility problems. It addresses patient-centered, organizational, and medical-technical issues on the clinical pathways, from first visiting the physician to completed treatment (with or without a pregnancy) and aftercare from the physician or medical psychologist (Table 3). The MuG consists of 198 recommendations based on the best available evidence or expert opinion; the level of evidence (A to D) is given for each recommendation. All recommendations were linked to the key questions formulated. Of these recommendations, 59% concerned organizational and patient-centered aspects of care (Table 4). The medical-technical recommendations for transitions in fertility care and supporting care alignment were derived from the MoGs. Twenty-one prioritized patients’ recommendations, obtained via WikiFreya, were included in the MuG and graded as level P evidence [18].

**Table 3:** Contents of the final version of the patient-centered multidisciplinary guideline for infertility

**Chapter 1: background**	Description of the patient-centered network approach
	Composition of the guideline development groups and methods used to involve patients
	Definition of infertility and description of patients’ clinical pathway
**Chapter 2: organization**	Organization of fertility care
	Registration of outcomes of infertility treatments
	Care alignment
**Chapter 3: physician**	Basic principles in fertility care for the physician
	History, physical examination, and additional infertility assessments
	Treatment policy
	Referral
	Information provision and education
	Coordination of primary care with secondary/tertiary care
	Attendant role after referral
**Chapter 4: gynecologist**	Basic principles of fertility care for the gynecologist
	History, physical examination, and additional infertility assessments
	Treatment policy
	Treatment criteria regarding age
	Information provision and education
	Referral
	Coordination of primary care with secondary/tertiary care
**Chapter 4: urologist**	Basic principles of fertility care for the urologist
	History, physical examination, and additional infertility assessments
	Treatment policy
	Coordination of primary care with secondary/tertiary care
**Chapter 5: semen analysis**	Basic principles for semen analysis
	Collection of semen
	Analyzing the semen
	Interpreting the results of a semen analysis
	Reporting the results
**Chapter 6: psychologist**	Basic principles in fertility care for the psychologist
	Psychological screening of patients with fertility problems
	Referral
**Chapter 7: sexologist**	
**Chapter 8: work and infertility**	Infertility in relation to occupation
	Exposure to harmful substances during work
	Participation of infertile patients in work
**Chapter 9: associations for fertility problems**	Opportunities and legislation for adoption

**Table 4:** Examples of recommendations integrated into the multidisciplinary guideline

**Medical-technical recommendations**	**Origin or guideline**	**Chapter of the MuG**
The physician should only physically examine the man if his semen analysis is irregular. *LOE C*	General infertility for physicians	Family physician (physical examination)
The gynecologist should not test ovarian reserve capacity to predict probability of pregnancy (with or without treatment). *LOE A*	Unexplained infertility	Gynecologist (assessments)
**Organizational recommendations**		
The physician should order a semen analysis from an accredited laboratory (ISO15189) or from a referral hospital. *LOE D*	MuG	Organization of fertility care
In accordance with the Dutch IVF planning decree, every licensed IVF center and their corresponding transport and satellite centers must provide annual reports on treatment outcomes for uniform national IVF registration. *LOE D*	MuG	Organization of fertility care (registration)
**Patient-centered recommendations**		
Both partners of the couple should be involved in the assessment and management of infertility because it is a joint problem. *LOE C*	MuG	Physician, gynecologist, and urologist (basic principles)
The gynecologist should offer couples with fertility problems psychological support throughout all phases of fertility care. *LOE D*	MuG	Gynecologist (information provision)
**Patient recommendations**		
Patients want their gynecologist to inform them about the different phases of treatment and their expected time spans. *LOE P*	MuG	Gynecologist (information provision)
Patients want their physician to make a referral immediately after they have been trying to conceive for 1 year. *LOE P*	MuG	physician (referral)

MuG = Multidisciplinary Guideline, IVF = in vitro fertilization, LOE = level of evidence.

### Time investments

The median number of two-hour regular group meetings was 10 (range: 5 to 11). The median participation rate was 88% (range: 77 to 94%). Three additional two-hour meetings were necessary for adjustment between guidelines. The steering committee needed 11 two-hour meetings for organizing the project.

Seven participants (20%) were involved in the preparation phase, for which they needed 471 h in total. In the development phase, the participants spent time on meeting preparation, two-hour face-to-face meetings, and minutes of meetings; and extra time on reviewing literature, writing guidelines, and commenting on draft versions. The time all participants spent in the development phase totaled 4,497 hours, including the 281 hours the steering committee members spent organizing this phase (Table 5).

**Table 5:** Time investments for phase 2: guideline development

**Guideline development groups and number of participants**	**Mean percentage of participation in *regular meetings *** **(number of meetings)**	**Median hours spent per participant in *regular meetings *** **(range)**	**Total hours**^**a **^**spent in *regular meetings *** **per development group**	**Total hours in *additional meetings *** **(number of participants)**	**Median extra hours per participant (range)**	**Total extra hours**^**b **^**per development group**	**Total hours**^**c **^**in the development phase**
MoG: general infertility; *n* = 7	92 (10)	40 (22.5–50)	258	7 (1)	58 (40–60)	348	**613**
MoG: unexplained infertility; *n* = 6	85 (10)	47 (18–72)	240	15 (3)	58 (50–60)	290	**545**
MoG: male infertility; *n* = 6	94 (8)	24 (12–56)	135	8 (2)	39 (18–50)	195	**338**
MoG: semen analysis; *n* = 5	88 (5)	30 (10–35)	132	-	48 (46–50)	192	**324**
MuG; *n* = 11	77 (11)	30 (21–55)	254	38 (10)	31 (2–51)	310	**602**
***Subtotal***			*1019*	*68*		*1335*	***2422***
Steering committee *n* = 7	87 (11)	25 (15–77)	152	39 (4)	15 (3–52)	90	**281**
*Project coordinator*^*c*^						*1794*^*c*^	***1794***^***c***^
**Total**			**1171**	**107**		**3219**	**4497**

MoG = Monodisciplinary guideline, MuG = multidisciplinary guideline.

*Regular meetings* are meetings necessary for the development of the guidelines.

*Additional meetings* are meetings necessary for discussing and refining the consistency of the guidelines.

*Extra time investments* are the hours participants needed to formulate key questions, review, select and assess evidence, write, review and revise draft guidelines, secure alignment of the guidelines and manage Wikifreya.

^*a*^Total hours for regular meetings are corrected for questionnaire non-responders (mean participation rate x number of meetings x median time investments).

^*b*^Total extra hours are corrected for questionnaire non-responders [(number of participants per development group-1) x median extra time per participant].

^*c*^The project coordinator’s meeting hours are included per development group; his extra hours are given separately.

### Feedback

#### Interviews

Most of the eight interviewees thought the guideline groups were well composed. Nevertheless, they perceived combining the role of moderating the meetings and providing clinical input for the content as unsatisfactory. The two project leaders said they underestimated the project coordinator’s workload, particularly in combining the coordination tasks with writing the draft guidelines. Furthermore, views and preferences differed between the chairperson of the MuG and the project coordinator about the scope, format, and content of the guideline; this formed a time-consuming barrier.

#### Questionnaires

The 27 questionnaires showed that most participants perceived the methodology of the network approach on the one hand as ‘the promising future of guideline development’ and on the other hand as ‘complex and unclear’. Positive notes included the perceived individual learning curves for guideline development, the opportunity of distributing key questions to participants with relevant knowledge, various participants’ perceived respect for autonomy, and the collaborative development of the one MuG. Furthermore, participants reported that the high level of coordination required to carry out the project as planned was a potential barrier to the approach. The different opinions among the professionals caused delays and tension in finalizing the guidelines. Clear expectations about the roles of the participants and a description of the final format for the guideline were lacking.

The integration of the MoGs into the MuG was seen as ‘powerful’, mainly regarding ‘special attention to transitions in different phases of care (alignment)’, ‘the opportunity to check possibly underexposed topics’ and ‘the simultaneous development of all guidelines’. The final equalization of guideline content was ‘too late’ (e.g., when recommendations had already been formulated), ‘difficult’ (due to differing opinions), and ‘time consuming’ (extra meetings). One respondent said integrating MoGs into the MuG was ‘needless’.

#### Patient involvement

All MuG group respondents described the participation of patient representatives in their group as ‘valuable’ (e.g., influencing discussions by refocusing on the patient) and their contributions as ‘beneficial to the final product’ (e.g., affecting formulations of considerations or expert-based recommendations). The representatives emphasized the need for information about the components of clinical care pathways before they discussed treatment options. More than one-half the respondents described the final patient recommendations as ‘valuable’ or ‘eye-openers’, and ‘useful’ in formulating professional recommendations. Some of them doubted the practical applicability of these recommendations. They questioned the fact that patients recommended that the physician immediately refer patients trying to conceive to the gynecologist. They noted the lack of new insights in patient recommendations.

#### Facilitators and barriers

Facilitators for the network approach included the selection of the most competent and dedicated participants, the introduction of the project coordinator, and patient contributions. Perceived political barriers, competing professional interests of those involved, and the lack of a more detailed MuG format created barriers. Suggestions for improvement included communication of clear instructions for individual roles and responsibilities, a strict schedule including deadlines, and a clear format for the guidelines. Further, the need for supportive staff and support for literature searches were noted.

## Discussion

This study provides detailed insight into the feasibility of a novel patient-centered network approach to MuG development for fertility care. This approach enabled the collaborative development of a harmonized set of one MuG and four MoGs for clinical care pathways for infertile couples. The approach helped us foresee possible barriers analogous to the US Institute of Medicine recommendations for developing trustworthy and transparent CPGs [9].

All the relevant stakeholders were included in the guideline groups. Collaboration between balanced groups of key stakeholders is an important success factor for clinical networks and may lead to a more valid method of developing guidelines [9,10]. A crossover of stakeholders from one guideline group to another helped harmonize the guideline content and distribute questions among the groups. This emphasized specific professional contributions and created a feeling of affinity with the guidelines. The development required intensive patient engagement, and the contributions of patient representatives in the MuG group and individual patients acting via WikiFreya were considered valuable [18].

The MuG follows patient clinical pathways and uses a network structure that includes all stakeholders, so that it pays much attention to the organization of the different phases of fertility care and transitions from one phase to another. This ensures better-integrated care (e.g., referral from the physician to the gynecologist) [22]. The attention to patient preferences, needs, and values may have increased the level of patient-centeredness [23]. The approach included an extensive review of the guidelines throughout the development. It used the network structure for which extra time was needed, but it enabled broader support of the guidelines and may enhance future guideline implementation [24,25].

The participants liked the approach and viewed it as a promising format for developing MuGs. Enthusiastic patients and the energetic project coordinator helped make the approach work well. Suggestions for improving the approach were reported mainly at the organizational level (e.g., previous communication about individual roles and responsibilities, a detailed time line, and a detailed format for the guidelines). This correlates with the existing literature about clinical networks, which implies that using clinical networks requires a high degree of managerial organization [13,14,22,26]. More support staff might enhance the efficiency of the network approach. Engaging a librarian to help with literature searches might accelerate guideline development and increase efficiency [27]. However, the approach seemed time consuming for developing our set of five related guidelines simultaneously. Unfortunately, it is difficult to compare our time investments with those of regular guideline development, since there is a dearth of published studies about this topic.

### Strengths and limitations

Although the use of a clinical network has been suggested as an effective strategy for implementing CPGs, this is the first study that has applied this approach in developing MuGs [28-31]. Our guideline development closely paralleled the main recommendations of the US Institute of Medicine [9]. However, recommendations were not graded and to express patients’ input, patients’ recommendations were secondarily reformulated to a non-actionable form incongruent to the GLIA instrument. This non-actionable form could impede harmonization of patients and stakeholders generated inputs. In addition to other studies on guideline development approaches, we also evaluated the feasibility of the approach. Nevertheless, a basis for comparing time data is lacking, which is a limitation of our study and a major limitation of current study designs of guideline development. In our opinion, guideline development is time consuming and expensive. Time should always be weighed against benefits, especially for introducing new approaches.

We have applied the patient-centered network approach to a MuG program for fertility care. This clinical area is characterized by the involvement of intensively collaborating professionals and responsible patients, which might be an argument against generalizability for other clinical areas. However, addressing practice change and sustaining clinical networks generally requires great motivation and is not specific to fertility care [32,33]. In this light, the level of our participants’ experience may have been a success factor in realizing the project in a relatively short time, but it may also be an argument against generalizability. Nevertheless, basic knowledge of guideline development methodologies is necessary in all approaches. Moreover, not only were our participants pretty experienced in guideline development, they were also opinion leaders within their own medical specialties. This may be an important success factor for disseminating and implementing the definitive guidelines. Despite this, we realize that, because our participants are rather experienced in their own usual way of developing guidelines, they might have been more critical of such a new approach. For instance, they regarded the lack of a detailed format as a barrier. This factor may have hindered the guideline development.

Although the guidelines clearly address organizational and patient-centered aspects (altogether, in 59% of the recommendations), we did not compare the proportion of these aspects to proportions in conventionally developed guidelines. However, we expect that the proportion of patient-centered aspects is rather small in other guidelines because patient participation in guideline development is still not common practice [34]. This mechanism may also apply to the organizational aspects, which are mainly addressed in guideline-related products, such as local protocols.

Although the participating member societies and organizations are committed to disseminating the final, harmonized guidelines, our detailed process evaluation was limited to the first phases of the guideline development and did not include the dissemination and implementation phases. We assume that this approach will enhance the implementation and our network might be an effective strategy in the further efforts that are still required [28-32,35].

### Conclusions and implications for further research

The novel approach of the patient-centered network is feasible for simultaneously and collaboratively developing a harmonized set of MuGs and MoGs for the clinical pathways of infertile couples. The approach is a potential strategy for developing more trustworthy and transparent guidelines. If consensus on the guideline format is reached beforehand, instructions on individual responsibilities within the network are provided, more support staff is employed, and a librarian is engaged to conduct systematic literature searches, then the network approach can be used in other guideline development programs too.

We believe that this approach may apply especially to patients who travel numerous complex pathways. Our study focuses on the network needed for patients who receive multidisciplinary fertility care and form the center of the network. However, other patients who travel different or multiple complex clinical pathways may also profit from this approach. Multiple networks can be connected or extended where necessary. However, this approach might be less valuable when patients travel a clear monodisciplinary pathway; for example, the pathway for a simple bone fracture. Further research is needed to compare the efficacy of this approach with more traditional approaches regarding content, time investments, and actual adoption of guidelines in a pragmatic randomized controlled trial.

### Ethics approval

Ethical approval was not required for this study. However, all participants gave informed consent before taking part. All observational data and transcripts of interviews were anonymized.

## Abbreviations

CPG: Clinical practice guideline; IVF: in vitro fertilization; MoG: Monodisciplinary guideline; MuG: Multidisciplinary guideline.

## Competing interests

All authors have completed the Unified Competing Interest form at http://www.icmje.org/coi_disclosure.pdf (available on request from the corresponding author) and declare: no support from any organization for the submitted work, no financial relationships with any organizations that might have an interest in the submitted work in the previous three years, and no other relationships or activities that could appear to have influenced the submitted work.

## Authors’ contributions

EB was a guarantor, helped design the study, analyze the data, interpret the analysis, and draft the paper. MH was a guarantor, helped design the study, conducted the in-depth interviews, and contributed to the questionnaire, the data analysis, interpretation of the analysis, and the draft of the paper. WN and TW helped design the study and draft the paper. JB helped interpret the analysis and draft the paper. JK was the principal investigator, and helped design the study and draft the paper. RH led the research team, contributed to the study design, the questionnaire, the data analysis, the analysis interpretation, and the drafting of the paper. All authors reviewed the consecutive drafts of the paper and gave their final approval of the version to be published.

## Authors’ information

Jean Vriezen and Jacintha van Balen from the Dutch College of General Practitioners, Utrecht, contributed to the study design, were members of the steering committee, and contributed to the revisions of the paper.
